# Integrated analysis of metabolome in a EUS-FNA sample with transcriptome in the TCGA cohort of pancreatic head and body/tail adenocarcinoma

**DOI:** 10.18632/aging.202700

**Published:** 2021-03-10

**Authors:** Chen Ke, Liu Yuan, Yang Xiujiang, Shen Danjie

**Affiliations:** 1 Department of Endoscopy, Fudan University Shanghai Cancer Center, Shanghai, China; 2 Department of Oncology, Shanghai Medical College, Fudan University, Shanghai, China; 3 Department of Digestive, Minhang Hospital, Fudan University, Shanghai, China

**Keywords:** pancreas, cancer, head, metabolome, endoscopic ultrasound

## Abstract

Metabolome profiles are largely unknown for pancreatic head cancers, in which the predominant anatomical feature is the exosure of bile, pancreatic juice, and duodenal juice. In this research, 30 head and 30 body/tail cytological samples acquired by endoscopic ultrasound-guided fine needle aspiration (EUS-FNA) of pancreatic adenocarcinoma were delivered for liquid chromatography coupled with mass spectrometry (LC-MS). Transcriptome analysis was performed using the sequencing data from The Cancer Genome Atlas (TCGA) cohort. LC-MS obtained 4,857 features in EUS-FNA cytological samples, and 586 metabolites were certified. Among them, 30 differential metabolites were identified. In the TCGA cohort, 247 differential metabolism genes were selected from 1,583 differential genes. The integrated analysis identified the top three enriched metabolic pathways as follows: branched chain amino acid (BCAA) biosynthesis; glycerophospholipid metabolism; and phenylalanine metabolism. In cell line, BCAA promoted pancreatic cancer proliferation and inhibited Oxaliplatin-induced apoptosis. In conclusion, metabolomic analysis with the EUS-FNA sample is feasible for pancreatic cancer. The integrated analysis can identify key metabolites and enzyme-coded genes between pancreatic head and body/tail adenocarcinoma. Anti-BCAA metabolism therapy may exert promising effect, especially for the body/tail cancer.

## INTRODUCTION

Pancreatic adenocarcinoma is the top ten leading cause of death from cancer in the world [1]. About 80% of pancreatic adenocarcinoma patients present with locally advanced findings or metastasis at the time of diagnosis, and the median survival time is less than 1 year. Surgical and the postoperative adjuvant therapies are only suitable for the remaining 20% patients, and the extended five-year survival rate is only 10% to 22%.

Several studies suggest that tumors behave differently with anatomical location, such as the cases of the left–right colorectal cancer and the greater–lesser curvature gastric cancer [2–4]. Tumor location implies a specific organic function, physiological feature, and histological constitution, and even genomic background. As for pancreatic cancer, data from the Surveillance, Epidemiology, and End Results database, Australian Pancreatic Cancer Genome Initiative cohort, and Dutch Pancreatic Cancer Group datasets have indicated higher incidence and better prognosis for head adenocarcinoma than for body/tail adenocarcinoma, but post-surgical prognosis remains to be the worse for the local stage [5–8].

The mechanism for the above disparity has been speculated based on the manifestation of the obstructive dilations of the bile and the pancreatic duct (i.e., jaundice and acute pancreatitis), and early detection seems to favor the management of pancreatic head cancer. However, when both types are resectable, the biological behavior at the body/tail location may be the same as that at the head location. Molecular classification based on large-scale genomic sequencing has been proposed to guide the precise and individual management of pancreatic cancer [9]. Dreyer and Birnbaum et al. demonstrated heterogeneity in the genomic background of different tumor locations [8, 10].

Pancreas is an organ with complex exocrine and endocrine functions. The predominate anatomical feature of pancreatic head cancer is the confluence of many kinds of metabolic agents, including bile, pancreatic juice, and duodenal juice. Elevated pressure due to obstruction by cancer triggers the reflux of juice and penetration into the microenvironment of cancer cells. Hence, identifying the metabolic response of pancreas to pathophysiological stimuli is of great value in reflecting the status of disease.

The research on the metabolome profile of pancreatic head and body/tail cancer with local samples is rare. Hence, in the current study, we first perform metabolome analysis with endoscopic ultrasound-guided fine needle aspiration (EUS-FNA) samples for the two groups. Then, the transcriptome profile is depicted based on The Cancer Genome Atlas (TCGA) cohort, an online and open genomic database of RNA sequences. Finally, the key metabolites and enzyme-coded genes between pancreatic head and body/tail adenocarcinoma are identified by integrated metabolome and transcriptome analysis.

## RESULTS

Sixty patients (mean age, 64.6±9.1 years; M/F, 38/22) undergoing EUS-FNA procedures were included in the study. The mean BMI was 22.2±2.9 mm. The dataset covered 30 pancreatic lesions in the head location and 30 lesions in the body/tail location. The mean diameter of lesions was 36.0±8.7 mm and ranged from 21.0 to 60.5 mm. In total, 40% cases were presented with dilated distal bile duct based on EUS or CT imaging. In the FNA process, the 22G-needle was used in 22 cases, whereas 25G-needle was used in 38 cases. All cases were eventually diagnosed as pancreatic adenocarcinoma. Table 1 shows that age, gender, lesion size, BMI, serum metabolism markers (Alb, BUN, LDH, FDG, and UA), and CA199 level were similar between the head group and the body/tail group without significant difference. However, the 22G-needle was more preferred for body/tail lesions than head lesions (*p*<0.001). The obstructive dilation of the bile duct was more common in the head group than in the body/tail group (*p*=0.001). The uniform epidemiological features and serum metabolism markers of the two groups indicate the similarity in their systemic metabolism levels.

**Table 1:** The clinical features of pancreatic cancer in head and body/tail.

	**Head (n=30)**	**Body/tail (n=30)**	**P-value**
Age (ys, mean±SD)	66.0 ± 7.6	63.2 ± 10.3	0.235
Gender (n, %)			1.000
Female	11 (36.7%)	11 (36.7%)	
Male	19 (63.3%)	19 (63.3%)	
BMI (mean±SD)	21.8 ± 3.0	22.6 ± 2.7	0.282
Lesion size (mm, mean±SD)	36.3 ± 6.6	35.8 ± 10.4	0.822
Needle Size (n, %)			<0.001
22G	1 (3.3%)	21 (70.0%)	
25G	29 (96.7%)	9 (30.0%)	
Serum Liver test			
Alb (g/L, mean±SD)	41.6 ± 4.4	43.1 ± 4.1	0.159
BUN (umol/L, mean±SD)	4.9 ± 1.5	4.7 ± 1.1	0.700
LDH (U/L, mean±SD)	169.4 ± 51.8	175.1 ± 64.4	0.707
FDG (mmol/L, mean±SD)	6.4 ± 2.6	6.8 ± 2.9	0.578
UA (umol/L, mean±SD)	252.0 ± 80.8	283.3 ± 62.4	0.098
Serum CA199 (n, %)			0.718
Normal	4 (13.3%)	5 (16.7%)	
Elevation	26 (86.7%)	25 (83.3%)	
Dilation of Bile duct (n, %)			0.001
Negative	19 (63.3%)	29 (96.7%)	
Positive	11 (36.7%)	1 (3.3%)	

**Figure 1:**
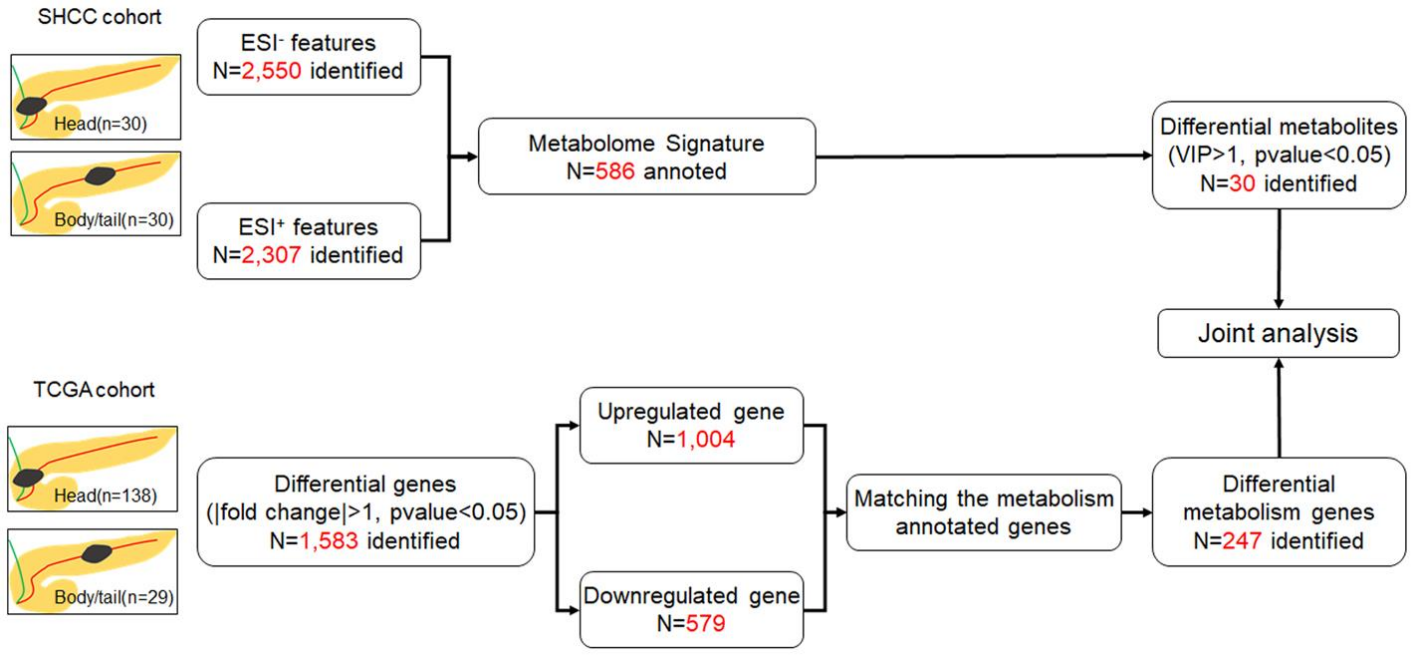
**Flow chart of the study.** The SHCC cohort and TCGA cohort were applied for metabolome and transcriptome analysis, respectively, and then joint analysis was performed. The pancreatic cancer was divided into head and body/tail groups, based on the anatomic location.

**Figure 2:**
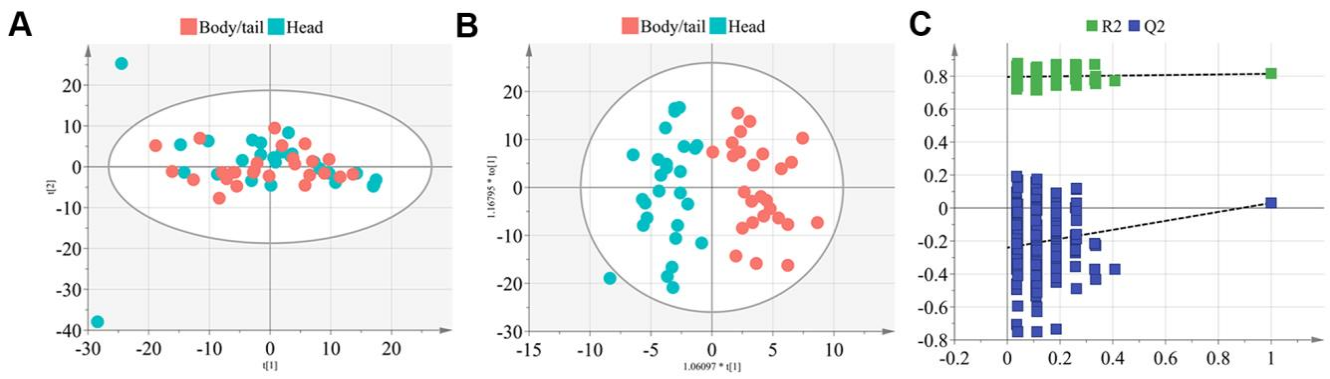
**The PCA and OPLS-DA model construction of metabolome signature.** (**A**) The plot depicted the distribution of the pancreatic head and body/neck cancers (n=30 for each group) into seven components with the PCA analysis (cum R^2^X=0.513, Q^2^=0.130), after removing the six outliers. (**B**) Deep mining by OPLS-DA identified an optimized classification (cum R^2^Y=0.818, Q^2^=0.031) into three components. (**C**) Results of the 200 times permutation test of the OPLS-DA model. The blue regression line of the Q2-points intersects the vertical axis below zero indicated the validity of the model.

All 60 samples were delivered for LC-MS analysis. In total, 2,550 features and 2,307 features were obtained at the electrospray ionization negative and positive (ESI^-^ and ESI^+^) ionic modes by LC-MS (Figure 1). In the metabolome analysis, we initially checked the experimental system by including the quality control samples into PCA. As shown in Supplementary Figure 1, all quality controls are clustered at the center of the coordinate axis, indicating the stability of the current experimental condition. Then, 586 metabolites were identified and annotated according to compound molecular weight and peak intensity. As shown in Supplementary Figure 2, the total PCA plot for the cum R^2^X is 0.583, and six outliers are apparent. After removing the outliers, seven components were derived from the updated PCA plot (cum R^2^X=0.513, Q^2^=0.130, Figure 2A). Deep mining by OPLS-DA identified an optimized classification (cum R^2^Y=0.818, Q^2^=0.031, Figure 2B) with three components. The results of the permutation analysis in Figure 2C prove that the model has good predictive ability, and no overfitting exists.

**Figure 3:**
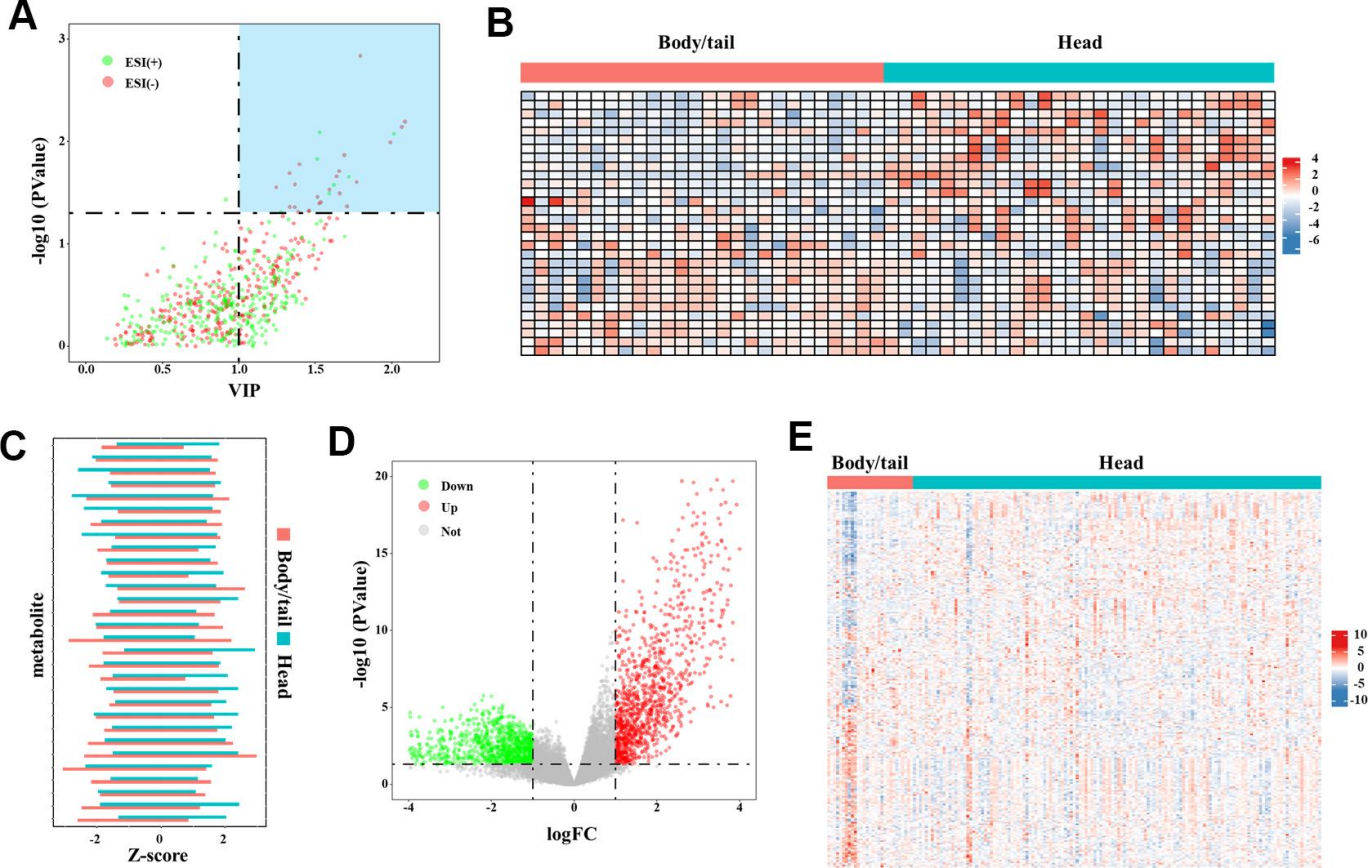
**The DEM and DMG identified in the metabolome and transcriptome data.** (**A**) The volcano plot of DEM denoted the selection of DEM (n=30), with VIP values >1 and p-value <0.05 provided from the t-test. The blue block in upper-right represented the selected DEM. (**B**) The heatmap (30◊60) depicted the Z-score of 30 DEM in metabolome analysis with log transformation, by setting body/tail cancers as referenced (n=30 for each group). (**C**) The bar chart depicted the direct comparison for each metabolite between pancreatic head and body/neck cancer. The Z-score with log transformation was presented. (**D**) Genes in the transcriptome analysis were presented in the volcano plot. By setting the fold-change >1 and p-value <0.05 as threshold, differential genes were depicted. The green point represented the downregulated genes (n=579) and the red points represented the upregulated genes (n=1,004). (**E**) The heatmap (247◊167) depicted the Z-score of DMG levels with log transformation, by setting body/tail cancers as referenced (n=138 for head group, and n=29 for body/tail group).

Thirty DEM are finally selected, with the filter of VIP set to greater than 1 and the p-value set to less than 0.05, as shown in the volcano plot in Figure 3A and Supplementary Table 1. The top five changed metabolites in descending order of VIP, were β-hydroxyisovaleric acid, L-isoleucine, methyl jasmonate, 3-carboxy-4-methyl-5-pentyl-2-furanpropanoic acid, and ketoleucine. The metabolite levels of the two groups are shown in the heat map and the bar chart Figure 3B, 3C, respectively. In the TCGA cohort, 1,583 differential genes can be identified, as shown in the volcano plot in Figure 3D. After matching the metabolism-annotated gene list, 247 differential metabolism genes were finally selected (Figure 3E).

**Figure 4:**
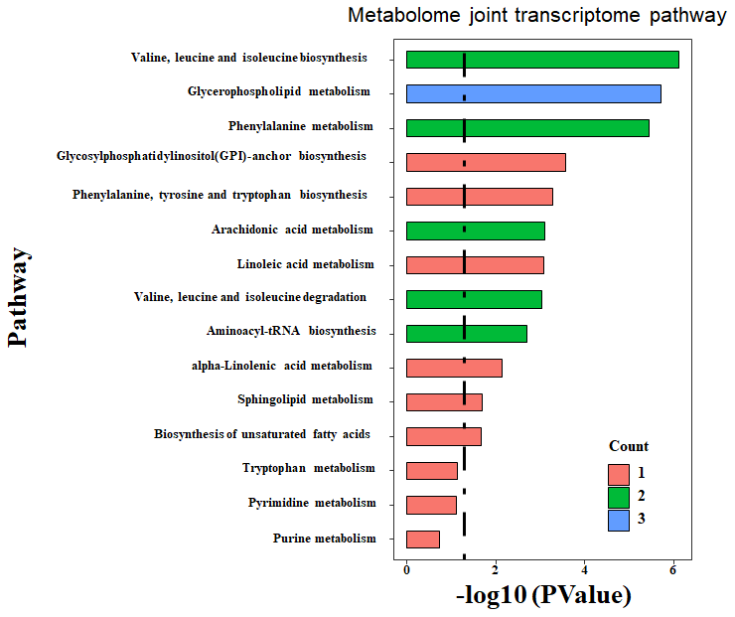
**Enriched pathway by integrated analysis.** Both the DEM and DEG were submitted to MetaboAnalyst, an online joint analysis module for integrated analysis. The count number is matched number of metabolites from the DEM with the pathway library. The more count number, the more reliable of the enriched pathway. The p-value is the statistical value from the over-representation analysis. The dashed line indicated the significant threshold p-value of 0.05.

**Figure 5:**
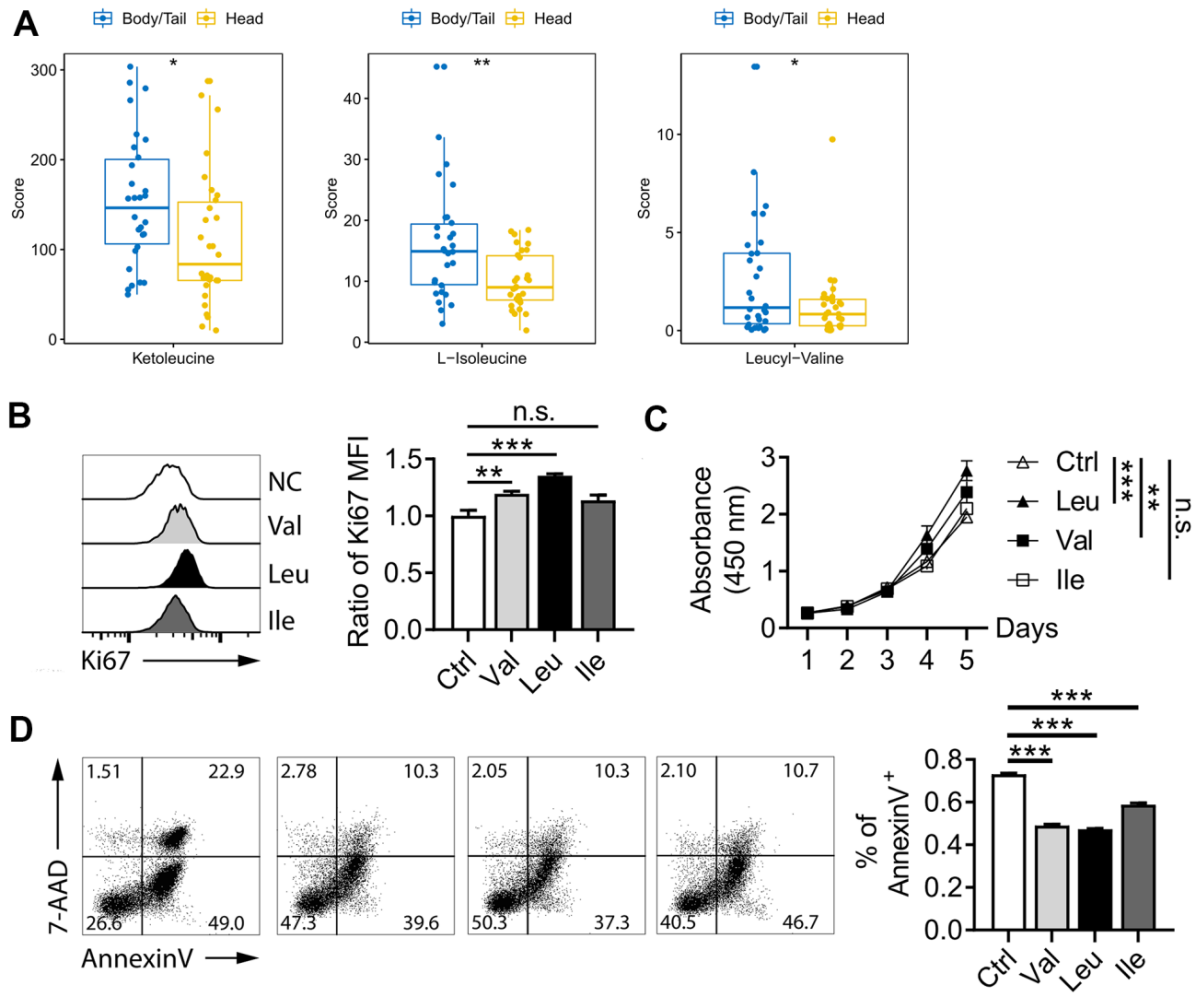
**BCAA pathway in pancreatic cancer.** (**A**) metabolites level of BCAA between pancreatic head and body/neck cancer (n=30 for each group). Val and Leu significantly promoted pancreatic cell line proliferation in Ki67 flow cytometry analysis (**B**) and CCK-8 test (**C**). Flow cytometry indicated Oxaliplatin treatment for 48 h deceased the rate of apoptosis (**D**). * p<0.05, ** p<0.005, *** p<0.001.

The joint enrichment analysis of metabolome and transcriptome data indicates that the top three differentially affected metabolism pathways between the two types of pancreatic cancer are BCAA biosynthesis (Leu, Ile, and Val) (*p*=0.002, impact=0.429), glycerophospholipid metabolism (*p*=0.003, impact=0.5), and phenylalanine metabolism (*p*=0.004, impact=0.6), as shown in Figure 4 and Table 2. The enriched metabolism maps are shown in Supplementary Figure 3. BCAA biosynthesis covers L-isoleucine and 4-methyl-2-oxopentanoate. Glycerophospholipid metabolism covers lysoPC (18:1(9Z)), PC (16:0/16:0), and PLA2G-, DGK-, GPD1L-, and PEMT-coded enzymes. Phenylalanine metabolism covers L-phenylalanine, 2-phenylacetamide, and DDC-, HPD-, and TAT-coded enzymes. The detailed metabolism level for BCAA was shown in Figure 5A.

**Table 2:** Integrated enrichment analysis of the metabolome and transcriptome data.

**Pathway**	**Total**	**Count**	**P-value**	**Impact**
Valine, leucine and isoleucine biosynthesis	8	2	0.002	0.429
Glycerophospholipid metabolism	34	3	0.003	0.500
Phenylalanine metabolism	11	2	0.004	0.600
Glycosylphosphatidylinositol-anchor biosynthesis	3	1	0.028	0.143
Phenylalanine, tyrosine and tryptophan biosynthesis	4	1	0.037	1.000
Arachidonic acid metabolism	37	2	0.045	0.472
Linoleic acid metabolism	5	1	0.046	0.250
Valine, leucine and isoleucine degradation	38	2	0.048	0.093
Aminoacyl-tRNA biosynthesis	46	2	0.067	0.044
alpha-Linolenic acid metabolism	13	1	0.117	0.083
Sphingolipid metabolism	21	1	0.182	0.550
Biosynthesis of unsaturated fatty acids	22	1	0.190	0.048
Tryptophan metabolism	40	1	0.320	0.051
Pyrimidine metabolism	41	1	0.327	0.083
Purine metabolism	68	1	0.485	0.014

To further vilify role of BCAA on pancreatic cancer, proliferation and Oxaliplatin-induced apoptosis were performed. As shown in Figure 5B, supplementary of Val and Leu significant promoted pancreatic cell line proliferation in Ki67 flow cytometry analysis, which also was proved by CCK-8 test (Figure 5C). While, no significant value was observed for Ile for both two experiments. In the Figure 5D, the rate of apoptosis as shown by the percentage of Annexin V^+^ positive cells was significant decreased after the Oxaliplatin treatment for 48 h. Those data indicated BCAA promoted pancreatic cancer proliferation and inhibited Oxaliplatin-induced apoptosis.

**Figure 6:**
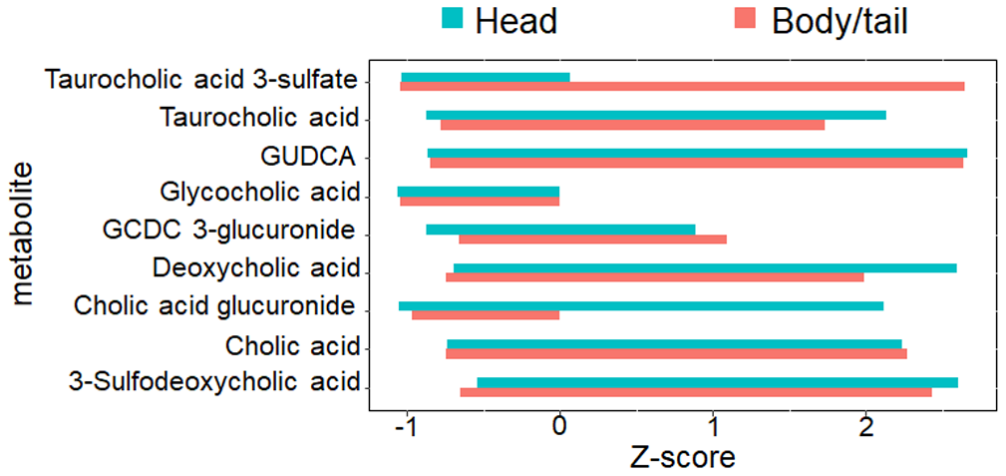
**The nine metabolites level involved with bile acid metabolism.** The bar chart denoted the direct comparison for the nine bile acid metabolites between pancreatic head and body/neck cancer (n=30 for each group). The Z-score with log transformation was presented. GUDCA, glycoursodeoxycholic acid, GCDC, glycochenodeoxycholic acid.

The obstructive dilation of the bile duct is common in head and thus may induce bile siltation in the microenvironment of the pancreatic cancer. Hence, bile acid metabolism was further checked in this study. As shown in Figure 6, no significant differences exist between the two groups for the nine metabolites (glycocholic acid, deoxycholic acid, cholic acid glucuronide, taurocholic acid, cholic acid, taurocholic acid 3-sulfate, glycoursodeoxycholic acid, glycochenodeoxycholic acid 3-glucuronide, and 3-sulfodeoxycholic acid) involved in bile acid metabolism.

## DISCUSSION

The current study initially investigated the metabolomic profiles of pancreatic head and body/tail cancer by using EUS-FNA-acquired cytological materials. Then, the transcriptome signature obtained by the TCGA cohort was integrated into the metabolome. Our findings indicate that the differential metabolites between head cancer and body/tail cancer are mainly enriched by the following: valine, leucine and isoleucine biosynthesis; glycerophospholipid metabolism; and phenylalanine metabolism. Bile acid metabolism was not involved with the pancreatic cancer of the head group.

Previous studies have reported the promising value of metabolome in identifying pancreatic cancer from non-malignant diseases, such as chronic pancreatitis [11–13]. Due to the specimen acquisition was easy and falsifiable, serum samples were collected for the metabolomics studies by different techniques, including gas chromatography coupled with mass spectrometry (GC-MS), LC-MS, or NMR spectroscopy. LC-MS is one of the most used techniques for untargeted metabolomic studies that have set higher sensitivities in finding the metabolite. However, a critical question is on whether the systemic perfused serum sample can specifically reflect the local cancer metabolomic profile. Trabado and Darst et al. reported that gender and age are the principal factors for plasma metabolome variability in healthy humans [14, 15]. The obesity measured by BMI has also been regarded a profound factor [16]. In addition, the uncontrolled bias from other unknown physiological and pathological conditions cannot be ignored in plasma metabolome. Nowadays, EUS-FNA almost is the routine examination in advanced pancreatic cancer for sample biopsy. EUS-FNA is the only minimally invasive technique for the "live" specimen acquisition. With the guidance of EUS, the specific tumor tissue can be targeted not only for routine cytohistological examination but also for next-generation sequencing [17–19]. Currently, no studies have been reported about the use of EUS-FNA samples for metabolomic analysis. However, Rezig et al. established a feasible metabolomic approach with the cytological material of thyroid tissues acquired by FNA via the 21-gauge needle [20]. In overcoming the flaw of serum sample, we initially performed EUS-FNA to acquire local cytological samples for metabolomic analysis. The PCA plot in our study has achieved stability in the current experimental condition for all quality controls clustered at the center of the coordinate axis. Hence, the EUS-FNA-based metabolome analysis is feasible for pancreatic cancer.

As we previously described, pancreatic head cancer suffers from more exposure to chemical agents, including bile, pancreatic juice, and duodenal juice, than body/tail cancer. Our findings reveal that BCAA biosynthesis, glycerophospholipid metabolism, and phenylalanine metabolism are the most enriched pathways. Amino acid metabolism is the most predominant pathway in pancreatic cancer. The BCAA, are linked with obesity, insulin resistance, and development of diabetes. Two prospective cohorts can robustly elucidate the predictive value of BCAA during pancreatic cancer development [21, 22]. Phenylalanine is also a member of aromatic amino acid (AAA) metabolism. No potential explanations have been reported for elevated BCAA biosynthesis and reduced phenylalanine metabolism pathways in pancreatic head cancer unlike in body/tail cancer. The concept of tissue context-directed activity of the amino acid metabolic pathway in cancer reported by Mayers et al. may offer insights for further investigation [23]. Our data shown the Leu, Ile, and Val were enriched in the body/tail adenocarcinoma, which shown a worse prognosis than head adenocarcinoma, reported on the previous clinical cohorts [5–8]. Consistently, in cell line, both three amino acid promoted pancreatic cancer proliferation and inhibited Oxaliplatin-induced apoptosis. Hence, those data indicated anti-BCAA metabolism therapy might exert promising effect, especially for the body/tail cancer.

Obstructive jaundice is the most common symptom of pancreatic head cancer due to the dilation of bile duct, and bile acid is the most widely investigated metabolic agent [24]. By contrast, for pancreatic body/tail cancer, the reflux of bile and duodenal juice is weak. *In vitro* experiments have proven the effect of bile acid on pancreatic acinar cell and ductal epithelium [25, 26]. However, direct evidence from exposure of pancreatic cancer to bile acid and metabolic profiling *in vivo* is lacking. Although the obstructive dilation of bile duct is more common in the head than in the body/tail, no difference for bile acid metabolites in the two tumor locations has been established in our study, which indicate that siltation of the bile does not always lead to the alteration of bile acid metabolism in the local region. We may also speculate that bile indirectly operates in the entire pancreas instead of directly stimulating the microenvironment. However, the enriched glycerophospholipid metabolism pathway in pancreatic head cancer may be associated with the siltation of the bile.

Studies have shown the value of metabolome in chemotherapy and chemoresistance [27, 28]. As for the chemotherapy of pancreatic cancer, no specific regimes are proposed for the head or body/tail group. And the chemoresistance is the major cause of the failure of therapy. The current study inspires us whether specific targets (such as the amino acid metabolism) exist for the drug discovery for different location of pancreatic cancer, which further research are warranted.

Limitations also exist in the current work. First, pancreatic head cancer and pancreatic body/tail cancer were investigated based on unpaired tumor samples and without the adjacent normal samples as reference. Considering a normal reference may benefit the identification of tumor specific metabolites. Second, the model classification and the selected DEM need to be validated by further external cohorts. Third, the metabolomic analysis has been restricted to advanced pancreatic cancer only. Extending our conclusions to the entire process of pancreatic carcinogenesis should be implemented carefully.

In conclusion, our data demonstrate that the EUS-FNA-acquired cytological sample is suitable for the metabolomic analysis of pancreatic cancer. Tumor location indeed plays a role in metabolic profiling. The integrated analysis indicates that amino acid pathways, including elevated BCAA biosynthesis and reduced phenylalanine metabolism, are enriched in head cancer. Anti-BCAA metabolism therapy might exert promising effect, especially for the body/tail cancer. The obstructive siltation of bile does not always lead to the enrichment of bile acid metabolites in pancreatic head, but it may be related to glycerophospholipid metabolism.

## MATERIALS AND METHODS

### Patients and sample collection

A total of 60 patients with suspected advanced pancreatic cancer underwent EUS-FNA from October 2018 to March 2019 at Fudan University Shanghai Cancer Center (SHCC) were consecutively included. The detailed clinical characteristics were retrieved from the database. All patients gave informed consent after approval of the current study by the institutional review board.

The detail of EUS-FNA procedure was described as before [29]. Two experienced endoscopists performed all the procedures. Linear longitudinal ultrasound endoscopes (EG3270UK and EG-3870UTK, Pentax, Japan) were applied under HI VISION ultrasound platform (Preirus HITACHI, Japan), with conscious sedation via intravenous midazolam and fentanyl. After inserting the needle into the echoendoscope, endoscopists repositioned the echoendoscope to set lesion at desired position and advanced needle. A 5-20 ml syringe was set to activate the suction. All the materials were finally delivered to the cytological and histological examination. Endoscopist directed rapid on-site evolution (ROSE) was performed in all cases during sample preparation. The remain cytological samples were flushed into tubes and stored at -80° C until use. Final diagnosis was defined by the histologic evidence in surgical, biopsy pathology, or definite cytology.

### Sample preparation

The aspirated sample (100 μL per case) was mixed with 300 μL of methanol, then vortexed for 30 s, and ultrasound for 20 min at ice bath. The sample was centrifuged at 12000 rpm for 15 min. The supernatant (200 μL) was transferred to vial for LC-MS analysis. Equal volumes (20 μL) of each sample were mixed and pooled into the quality control sample.

### LC-MS analysis

The workflow was shown in Figure 1. All experiments were performed on an Ultimate 3000LC platform (Thermo Scientific, USA) and equipped with Hyper gold C18 (100x2.1mm 1.9 μm) column. Chromatographic separation conditions included column temperature at 40° C, flow rate at 0.35 mL/min, automatic injector temperature at 4° C, injection volume at 10 μL. The mobile phase A was water plus 5 % acetonitrile and 0.1% formic acid. The mobile phase B was acetonitrile plus 0.1% formic acid. Metabolomics feature extraction and preprocessed were performed with Compound Discoverer software (Thermo Scientific, USA). The raw data was normalized and edited into two-dimensional data matrix by excel 2010 software, including retention time, compound molecular weight, samples, and peak intensity.

### Metabolome joint transcriptome pathway

The metabolome data mining was carried out by SIMCA (version 14.1). Unsupervised principal components analysis (PCA) and orthogonal partial least squares discriminant analysis (OPLS-DA) were performed to construct discriminated models for head and body/tail cancers. R^2^Y and Q^2^ were the evaluated parameters, which represented the fitness and prediction ability of OPLS-DA model. A permutation test with 200 times was examined to certify the model’s validity. The unpaired student’s t-test was used to compare the difference between two groups. The differential metabolites (DEM) were selected and ranked by variable importance in the projection (VIP) values >1 and p-value <0.05 provided from the t-test.

Genomic data of RNA sequencing for pancreatic carcinoma was queried from TCGA database. To investigate the genomic profile between head and body/tail group, edgeR Package [30] was applied to select the differential genes with fold-change >1 and p-value <0.05 in TCGA cohort. Then, differential metabolism genes (DMG) were identified and mapped in a previous 2,752 metabolism-annotated genes list [31].

The integrated analysis was performed by joint analysis module in MetaboAnalyst [32]. Over-representation analysis based on hypergeometric analysis was applied for enrichment. The topology analysis was based on the degree centrality within a pathway.

### Cell lines and culture

Human pancreatic cancer cell line Panc-1 was were purchased from the Cell Bank of Type Culture Collection of Chinese Academy of Sciences, and have been authenticated by Chinese Academy of Sciences and cultured in RPMI-1640 medium (Gibco, CA, USA). Cells were incubated in a humidified atmosphere at 37° C with 5% CO2. The RPMI-1640 medium included leucine (Leu, 50mg/L), isoleucine (Ile, 50mg/L), and valine (Val, 20mg/L), which was termed as control medium. The branched chain amino acid (BCAA) medium was supplemented with purified amino acids (Sigma) to 10x leucine (500mg/L), 10x Ile (500mg/L), or 10x Val (200mg/L), respectively.

### Cell proliferation and apoptosis detection

Flow cytometry was used to evaluate cell proliferation and apoptosis. Incubation with BCAA medium for 48h, Panc-1 cell was harvested and labeled with Ki-67-FITC (Biolegend) for proliferation detection.

Panc-1 cells (2×10^3^/well) was suspended in 100 μL RPMI-1640 medium and incubated in 96-well plates. After cell adherence, different medium was added, and culture for five days. The cell proliferation rate was calculated by cell counting kit-8 (CCK-8) regent (Dojindo Laboratories), according to the protocol.

Oxaliplatin (Selleck) induced cell apoptosis was detected. Panc-1 cells (2×10^6^/well) was incubated with Oxaliplatin (10 μmol/L) for 48 h, and harvested for flow cytometry by labelling Annexin V-FITC (Biolegend), and 7-AAD (Biolegend). Annexin V +/7-AAD - were early apoptotic cells and Annexin V +/7-AAD + were late apoptotic cells. The percentage of both early and late apoptosis were calculated.

Flow cytometry was performed using a BD FACS Canto II flow cytometer (BD Biosciences, San Diego, CA, USA). Data were analyzed using the FlowJo software (Flowjo, Treestar Inc., Ashland, OR, USA).

### Statistical

Categorical variables were compared by using Chi-square tests, and continuous variables were compared by using unpaired student’s t-test. A P value < 0.05 was considered statistically significant. All statistical analyses were performed using the SAS 8.02 software package (SAS Institute, USA).

## Supplementary Material

Supplementary Figures

Supplementary Table 1

## References

[1] Karakas Y, Lacin S, Yalcin S. Recent advances in the management of pancreatic adenocarcinoma. Expert Rev Anticancer Ther. 2018; 18:51–62. 10.1080/14737140.2018.1403319 29125367

[2] Petrelli F, Tomasello G, Borgonovo K, Ghidini M, Turati L, Dallera P, Passalacqua R, Sgroi G, Barni S. Prognostic survival associated with left-sided vs right-sided colon cancer: a systematic review and meta-analysis. JAMA Oncol. 2017; 3:211–19. 10.1001/jamaoncol.2016.4227 27787550

[3] Jung YJ, Seo HS, Kim JH, Park CH, Lee HH. Cross-sectional location of gastric cancer affects the long-term survival of patients as tumor invasion deepens. Ann Surg Oncol. 2017; 24:3947–53. 10.1245/s10434-017-6101-2 28980179

[4] Deng K, Han P, Song W, Wang Z, Zhang F, Xie H, Zhao W, Xu H, Cai Y, Rong Z, Yu X, Cui BB, Li K. Plasma metabolomic profiling distinguishes right-sided from left-sided colon cancer. Clin Chim Acta. 2018; 487:357–62. 10.1016/j.cca.2018.10.010 30296444

[5] Artinyan A, Soriano PA, Prendergast C, Low T, Ellenhorn JD, Kim J. The anatomic location of pancreatic cancer is a prognostic factor for survival. HPB (Oxford). 2008; 10:371–76. 10.1080/13651820802291233 18982154 PMC2575681

[6] Lau MK, Davila JA, Shaib YH. Incidence and survival of pancreatic head and body and tail cancers: a population-based study in the United States. Pancreas. 2010; 39:458–62. 10.1097/MPA.0b013e3181bd6489 19924019

[7] van Erning FN, Mackay TM, van der Geest LG, Groot Koerkamp B, van Laarhoven HW, Bonsing BA, Wilmink JW, van Santvoort HC, de Vos-Geelen J, van Eijck CH, Busch OR, Lemmens VE, Besselink MG, and Dutch Pancreatic Cancer Group. Association of the location of pancreatic ductal adenocarcinoma (head, body, tail) with tumor stage, treatment, and survival: a population-based analysis. Acta Oncol. 2018; 57:1655–62. 10.1080/0284186X.2018.1518593 30264642

[8] Dreyer SB, Jamieson NB, Upstill-Goddard R, Bailey PJ, McKay CJ, Biankin AV, Chang DK, and Australian Pancreatic Cancer Genome Initiative. Defining the molecular pathology of pancreatic body and tail adenocarcinoma. Br J Surg. 2018; 105:e183–91. 10.1002/bjs.10772 29341146 PMC5817249

[9] Bailey P, Chang DK, Nones K, Johns AL, Patch AM, Gingras MC, Miller DK, Christ AN, Bruxner TJ, Quinn MC, Nourse C, Murtaugh LC, Harliwong I, et al, and Australian Pancreatic Cancer Genome Initiative. Genomic analyses identify molecular subtypes of pancreatic cancer. Nature. 2016; 531:47–52. 10.1038/nature16965 26909576

[10] Birnbaum DJ, Bertucci F, Finetti P, Birnbaum D, Mamessier E. Head and body/tail pancreatic carcinomas are not the same tumors. Cancers (Basel). 2019; 11:497. 10.3390/cancers11040497 30965637 PMC6520848

[11] Bathe OF, Shaykhutdinov R, Kopciuk K, Weljie AM, McKay A, Sutherland FR, Dixon E, Dunse N, Sotiropoulos D, Vogel HJ. Feasibility of identifying pancreatic cancer based on serum metabolomics. Cancer Epidemiol Biomarkers Prev. 2011; 20:140–47. 10.1158/1055-9965.EPI-10-0712 21098649

[12] Mayerle J, Kalthoff H, Reszka R, Kamlage B, Peter E, Schniewind B, González Maldonado S, Pilarsky C, Heidecke CD, Schatz P, Distler M, Scheiber JA, Mahajan UM, et al. Metabolic biomarker signature to differentiate pancreatic ductal adenocarcinoma from chronic pancreatitis. Gut. 2018; 67:128–37. 10.1136/gutjnl-2016-312432 28108468 PMC5754849

[13] Lindahl A, Heuchel R, Forshed J, Lehtiö J, Löhr M, Nordström A. Discrimination of pancreatic cancer and pancreatitis by LC-MS metabolomics. Metabolomics. 2017; 13:61. 10.1007/s11306-017-1199-6 28413374 PMC5376388

[14] Trabado S, Al-Salameh A, Croixmarie V, Masson P, Corruble E, Fève B, Colle R, Ripoll L, Walther B, Boursier-Neyret C, Werner E, Becquemont L, Chanson P. The human plasma-metabolome: reference values in 800 French healthy volunteers; impact of cholesterol, gender and age. PLoS One. 2017; 12:e0173615. 10.1371/journal.pone.0173615 28278231 PMC5344496

[15] Darst BF, Koscik RL, Hogan KJ, Johnson SC, Engelman CD. Longitudinal plasma metabolomics of aging and sex. Aging (Albany NY). 2019; 11:1262–82. 10.18632/aging.101837 30799310 PMC6402508

[16] Cirulli ET, Guo L, Leon Swisher C, Shah N, Huang L, Napier LA, Kirkness EF, Spector TD, Caskey CT, Thorens B, Venter JC, Telenti A. Profound perturbation of the metabolome in obesity is associated with health risk. Cell Metab. 2019; 29:488–500.e2. 10.1016/j.cmet.2018.09.022 30318341 PMC6370944

[17] Rodriguez SA, Impey SD, Pelz C, Enestvedt B, Bakis G, Owens M, Morgan TK. RNA sequencing distinguishes benign from Malignant pancreatic lesions sampled by EUS-guided FNA. Gastrointest Endosc. 2016; 84:252–58. 10.1016/j.gie.2016.01.042 26808815

[18] Wang X, Gao J, Ren Y, Gu J, Du Y, Chen J, Jin Z, Zhan X, Li Z, Huang H, Lv S, Gong Y. Detection of KRAS gene mutations in endoscopic ultrasound-guided fine-needle aspiration biopsy for improving pancreatic cancer diagnosis. Am J Gastroenterol. 2011; 106:2104–11. 10.1038/ajg.2011.281 21876563

[19] Elhanafi S, Mahmud N, Vergara N, Kochman ML, Das KK, Ginsberg GG, Rajala M, Chandrasekhara V. Comparison of endoscopic ultrasound tissue acquisition methods for genomic analysis of pancreatic cancer. J Gastroenterol Hepatol. 2019; 34:907–13. 10.1111/jgh.14540 30422342 PMC6497552

[20] Rezig L, Servadio A, Torregrossa L, Miccoli P, Basolo F, Shintu L, Caldarelli S. Diagnosis of post-surgical fine-needle aspiration biopsies of thyroid lesions with indeterminate cytology using HRMAS NMR-based metabolomics. Metabolomics. 2018; 14:141. 10.1007/s11306-018-1437-6 30830426

[21] Mayers JR, Wu C, Clish CB, Kraft P, Torrence ME, Fiske BP, Yuan C, Bao Y, Townsend MK, Tworoger SS, Davidson SM, Papagiannakopoulos T, Yang A, et al. Elevation of circulating branched-chain amino acids is an early event in human pancreatic adenocarcinoma development. Nat Med. 2014; 20:1193–98. 10.1038/nm.3686 25261994 PMC4191991

[22] Katagiri R, Goto A, Nakagawa T, Nishiumi S, Kobayashi T, Hidaka A, Budhathoki S, Yamaji T, Sawada N, Shimazu T, Inoue M, Iwasaki M, Yoshida M, Tsugane S. Increased levels of branched-chain amino acid associated with increased risk of pancreatic cancer in a prospective case-control study of a large cohort. Gastroenterology. 2018; 155:1474–82.e1. 10.1053/j.gastro.2018.07.033 30076838

[23] Mayers JR, Torrence ME, Danai LV, Papagiannakopoulos T, Davidson SM, Bauer MR, Lau AN, Ji BW, Dixit PD, Hosios AM, Muir A, Chin CR, Freinkman E, et al. Tissue of origin dictates branched-chain amino acid metabolism in mutant Kras-driven cancers. Science. 2016; 353:1161–65. 10.1126/science.aaf5171 27609895 PMC5245791

[24] Feng HY, Chen YC. Role of bile acids in carcinogenesis of pancreatic cancer: an old topic with new perspective. World J Gastroenterol. 2016; 22:7463–77. 10.3748/wjg.v22.i33.7463 27672269 PMC5011662

[25] Perides G, Laukkarinen JM, Vassileva G, Steer ML. Biliary acute pancreatitis in mice is mediated by the G-protein-coupled cell surface bile acid receptor Gpbar1. Gastroenterology. 2010; 138:715–25. 10.1053/j.gastro.2009.10.052 19900448 PMC2819588

[26] Joshi S, Cruz E, Rachagani S, Guha S, Brand RE, Ponnusamy MP, Kumar S, Batra SK. Bile acids-mediated overexpression of MUC4 via FAK-dependent c-jJun activation in pancreatic cancer. Mol Oncol. 2016; 10:1063–77. 10.1016/j.molonc.2016.04.007 27185392 PMC4972654

[27] Navarrete A, Armitage EG, Musteanu M, García A, Mastrangelo A, Bujak R, López-Casas PP, Hidalgo M, Barbas C. Metabolomic evaluation of mitomycin C and rapamycin in a personalized treatment of pancreatic cancer. Pharmacol Res Perspect. 2014; 2:e00067. 10.1002/prp2.67 25505613 PMC4186447

[28] Armitage EG, Ciborowski M. Applications of metabolomics in cancer studies. Adv Exp Med Biol. 2017; 965:209–34. 10.1007/978-3-319-47656-8_9 28132182

[29] Liu Y, Chen K, Yang XJ. Endoscopic ultrasound-guided fine-needle aspiration used in diagnosing gastric linitis plastica: metastatic lymph nodes can be valuable targets. J Gastroenterol Hepatol. 2019; 34:202–06. 10.1111/jgh.14300 29864202

[30] Robinson MD, McCarthy DJ, Smyth GK. edgeR: a Bioconductor package for differential expression analysis of digital gene expression data. Bioinformatics. 2010; 26:139–40. 10.1093/bioinformatics/btp616 19910308 PMC2796818

[31] Nwosu ZC, Megger DA, Hammad S, Sitek B, Roessler S, Ebert MP, Meyer C, Dooley S. Identification of the Consistently Altered Metabolic Targets in Human Hepatocellular Carcinoma. Cell Mol Gastroenterol Hepatol. 2017; 4:303–23.e1. 10.1016/j.jcmgh.2017.05.004 28840186 PMC5560912

[32] Chong J, Soufan O, Li C, Caraus I, Li S, Bourque G, Wishart DS, Xia J. MetaboAnalyst 4.0: towards more transparent and integrative metabolomics analysis. Nucleic Acids Res. 2018; 46:W486–94. 10.1093/nar/gky310 29762782 PMC6030889

