# Supplementary material for: Integrated analysis of metabolome in a EUS-FNA sample with transcriptome in the TCGA cohort of pancreatic head and body/tail adenocarcinoma
Source: Aging (Albany NY). 2021 Mar 10;13(6):8880–94. doi: 10.18632/aging.202700 (PMC8034907; doi:10.18632/aging.202700)
Supplement: Supplementary Figures [file aging-13-202700-s001.pdf]

SUPPLEMENTARY FIGURES

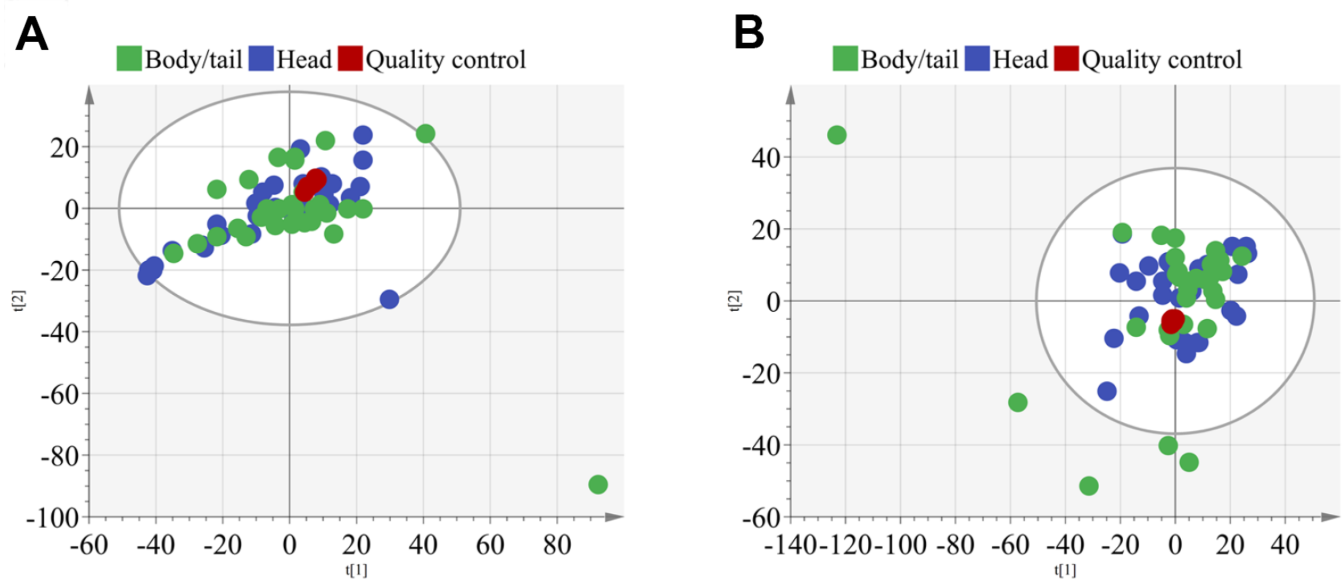

Supplementary Figure 1. The quality control testing by including quality control samples into the PCA analysis. (**A**) the PCA plot in ESI^+ mode. (**B**) the PCA plot in ESI^- mode.

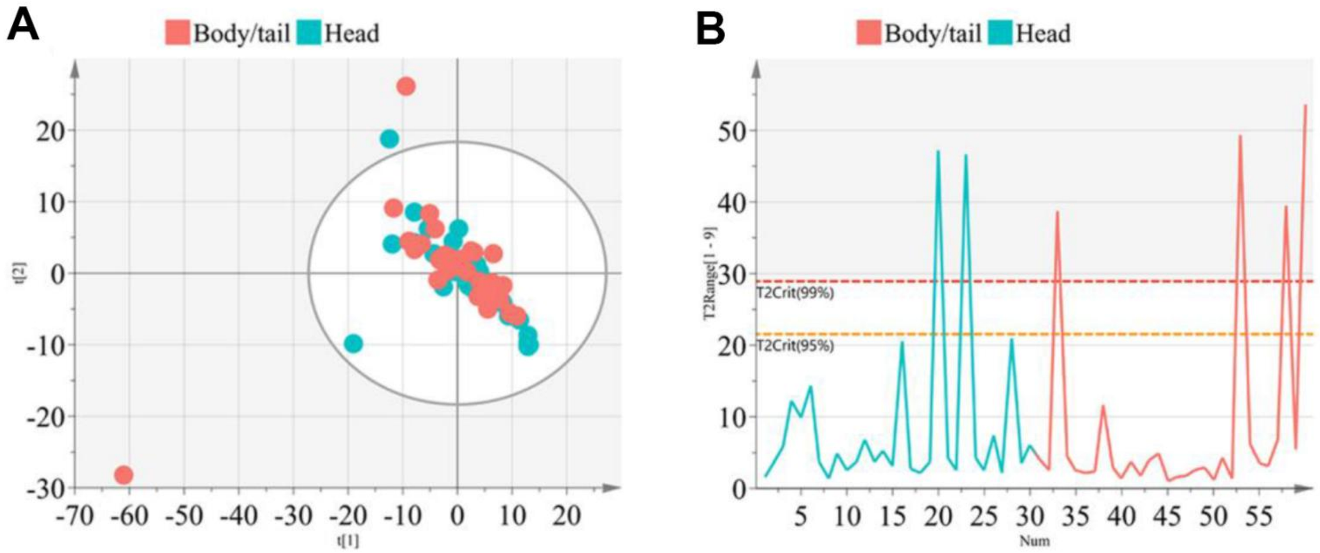

Supplementary Figure 2. The total PCA analysis indicated the six outliers. (**A**) the PCA plot. (**B**) Hotelling's T2 plot shows six cases with values larger than the 99% confidence limit were considered as outlier.

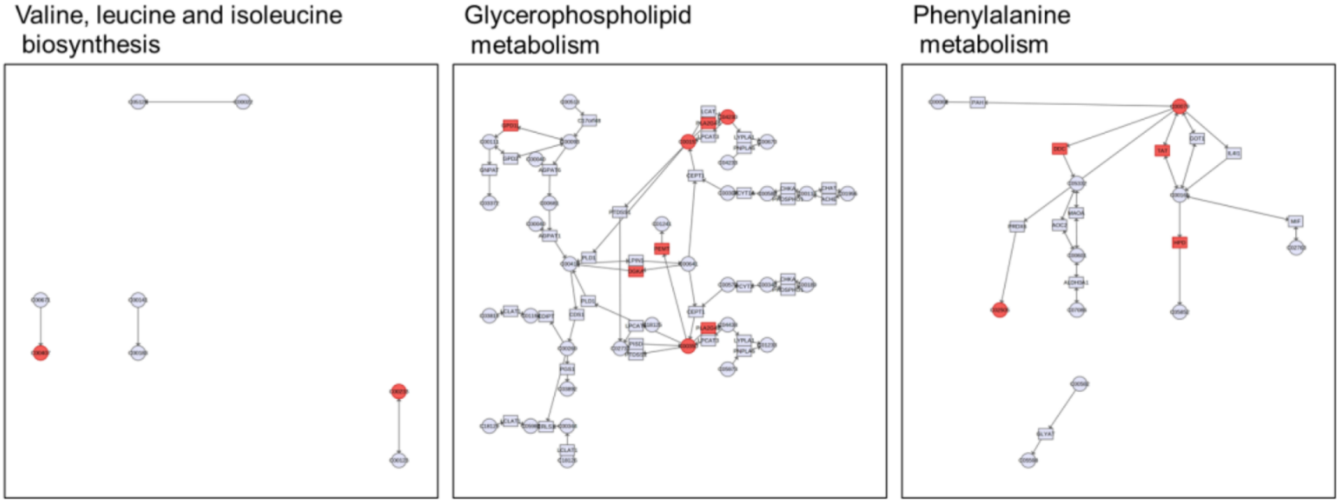

Supplementary Figure 3. The metabolism pathway of the top three enriched items, labeled with metabolites and enzyme-coded genes.
